# Transport of deoxy-d-glucose into lymphocytes of patients with polycystic ovary syndrome

**DOI:** 10.1007/s12020-014-0174-5

**Published:** 2014-02-11

**Authors:** Bożenna Oleszczak, Leszek Szablewski, Monika Pliszka, Olgierd Głuszak, Urszula Stopińska-Głuszak

**Affiliations:** 1 Chair of General Biology and Parasitology, Center for Biostructure Research, Medical University of Warsaw, 5 Chalubinskiego Str, 02-004 Warsaw, Poland; 2 Department of Endocrinology, Medical Centre for Postgraduate Education, Bielanski Hospital, 80 Cegłowska Str, 01–809 Warsaw, Poland

**Keywords:** Glucose transport, GLUT4, Lymphocytes, Insulin resistance, Diabetes

## Abstract

Polycystic ovary syndrome (PCOS) is linked to increased risk of insulin resistance and diabetes mellitus in patients’ later life. The aim of this study was to investigate uptake of deoxy-d-glucose by peripheral blood lymphocytes of PCOS patients with normal fasting plasma glucose and normal glucose tolerance test. The study involved 20 patients with PCOS with normal fasting plasma glucose and normal glucose in 60 and 120 min of oral glucose tolerance test, aged 18–32 (mean 23), BMI between 20 and 30 (mean 26). A control group consisted of 20 healthy women matched for glucose level (normoglycemia), aged 18–28 years (mean 23), BMI 20–25 (mean 23). Blood for the studies was collected in fasting conditions onto heparin. Lymphocytes were isolated within 2 h from collection by centrifuging. The intracellular transport into lymphocytes was studied using tritium-labeled deoxy-d-glucose and measured with a liquid scintillation counter. Radioactivity in curie per minute (cpm) was evaluated after 24 h. An initial examination was performed to check the presence of GLUT4 in peripheral blood lymphocytes of PCOS women. In all of the studied time points, the value of cpm for lymphocytes of PCOS patients was statistically significantly lower in comparison with the value obtained for lymphocytes of healthy women. However, the profile of deoxy-d-glucose uptake (d cpm) was the same for lymphocytes in both studied groups without statistically significant differences. In lymphocytes of PCOS patients, GLUT4 was detected. The obtained results indicate that PCOS affects the intracellular transport of deoxy-d-glucose into lymphocytes of PCOS patients with normal glucose level.

## Introduction

Polycystic ovary syndrome (PCOS) is a common endocrine disorder affecting ~ 6–10 % of women in reproductive age [3, 21]. It is also a major cause of menstrual disturbances, hirsutism, and infertility due to chronic anovulation. According to some studies, PCOS patients experience increased risk of developing diabetes mellitus [10, 11, 22], hypertension [36], dislipidemia [35], and atherosclerosis [4, 20]. These factors are usually present even among younger women [14]. Epidemiological data suggest that around 38–88 % of women with PCOS are overweight or obese [5, 38]. It is well known that visceral obesity carries increased risk for insulin resistance, a key factor in the pathophysiology of PCOS [1, 27].

 Insulin resistance influences glucose metabolism. In peripheral blood cells, GLUT proteins are responsible for transporting glucose. GLUT1, GLUT3, and GLUT6 are typical glucose transporters for lymphocytes and are primarily responsible for glucose transport into resting lymphocytes [13, 17, 23]. GLUT4 is typical for muscle and adipocyte cells [6, 7] and is insulin responsive. Insulin induces GLUT4 translocation from intracellular compartment into cell membrane. As a result, it rapidly increases cells’ uptake of glucose, especially in muscles and fat tissue [8, 28]. The increase of glucose uptake was observed in lymphocytes of type 2 diabetes patients treated with insulin [34]. In the resting state, only small percent of B and T lymphocytes demonstrated GLUT4 on the plasma membrane [26]. In the presence of insulin, B lymphocytes increase the abundance of GLUT4 on the plasma membrane; in contrast, T lymphocytes (dominant subpopulation of lymphocytes in the peripheral blood) are unresponsive to insulin [23].

Peripheral blood lymphocytes seem to be well-suited for studies on glucose homeostasis. It has been revealed that intracellular transport of deoxy-d-glucose in isolated lymphocytes is impaired among patients suffering from type 2 diabetes. Qualitative and quantitative changes in glucose transport and expression of glucose transporters were found in these cells in all diabetic patients, independent of the mode of therapy [31]. Also, different glucose concentrations (equal to hypoglycemia and hyperglycemia) in medium impair the uptake of deoxy-d-glucose by lymphocytes [26].

We have mentioned in the Introduction that PCOS is connected with increased risk of diabetes mellitus. Type 1 diabetes and type 2 diabetes are associated with pathological disorders in glucose transport. Therefore, it seems interesting to determine whether any changes in glucose transport into lymphocytes obtained from women with both PCOS and normoglycemia can be observed.

The aim of this study was to investigate whether PCOS as a disease influences intracellular transport of deoxy-d-glucose in peripheral blood lymphocytes of patients with normal fasting plasma glucose and normal glucose tolerance test.

Additionally, an initial examination was performed to check the presence of GLUT4 in peripheral blood lymphocytes of PCOS women as the amount of this glucose transporter is increased in lymphocytes of diabetic patients in comparison with healthy subjects [33].

## Materials and methods

### Patients

The patients who participated in the study were recruited from Department of Endocrinology of Bielanski Hospital in Warsaw. From 93 PCOS patients, only 20 who had normal fasting plasma glucose as well as normal glucose in 60 and 120 min of oral glucose tolerance test have been selected for the study.

The diagnosis of PCOS was based on the ESHRE/ASRM criteria [29] and required two of the following three features: menstrual disorders (oligomenorrhoea or amenorrhea), clinical or biochemical evidence of hyperandrogenism, and polycystic ovaries visible on ultrasound examination (at least 10 follicles measuring 2–9 mm or volume of the ovary > 10 cm^3^). The exclusion criteria were the following: presence of other endocrinopathy such as diabetes mellitus, Cushing’s syndrome, hyperprolactinemia, and adrenal hyperplasia or hormone therapy within past three months. All patients had the ultrasound examinations performed in the early follicular phase of the menstrual cycle. The physical examination included the following: anthropometric measurements (height, weight, body mass index −26.03 ± 3.1), menstrual history, presence of acne, hirsutism. Parameters measured were the following: fasting insulin (9.3 ± 5.4 μU/mL), fasting plasma glucose (82.52 ± 9.29 mg/dL), plasma glucose level, and insulin level in the oral glucose tolerance test (OGTT) with 75 g of glucose (glucose in 60 min of OGTT −104.5 ± 36.5 mg/dL, in 120 min −93.3 ± 23.4 mg/dL; insulin in 60 min of OGTT −46.5 ± 42 μU/mL, in 120 min −50.3 ± 43.9 μU/mL). Characteristics of women with PCOS who participated in the study are presented in Table 1.

**Table 1:** Characteristics of the PCOS patients participated in the study

n	20
Age (years)	23.7 ± 5.9
BMI (kg/m^2^)	26.03 ± 3.1
BMI 20–25	50 % (*n* = 10)
BMI 25–30	45 % (*n* = 9)
BMI > 30	5 % (*n* = 1)
Fasting insulin (μU/mL)	9.3 ± 5.4
Insulin after 60 min of OGTT (μU/mL)	46.5 ± 42.2
Insulin after 120 min of OGTT (μU/mL)	50.3 ± 43.9
Fasting blood glucose (mg/dL)	82.52 ± 9.29
Fasting blood glucose (mmol/L)	4.6 ± 0.52
Glucose in 60 min of OGTT (mg/dL)	104.5 ± 36.5
Glucose in 60 min of OGTT (mmol/L)	5.8 ± 2.03
Glucose in 120 min of OGTT (mg/dL)	93.3 ± 23.4
Glucose in 120 min of OGTT (mmol/L)	5.2 ± 1.3
HOMA-IR index	1.99 ± 1.17
QUICK index	0.35 ± 0.03
GLUT4 detected	in 80 % (*n* = 16)
GLUT4 not detected	in 20 % (*n* = 4)

Regrettably, the gold standard of hyperinsulinemic euglycemic clamp involves high costs and difficulties in execution [2]. Thus, for assessing insulin resistance, we have decided to use homeostatic model assessment–insulin resistance (HOMA-IR) as well as quantitative insulin sensitivity check (QUICK) indices. These indices are simple and minimally invasive. QUICK index is a variation of HOMA equations and is thought to be a consistent and precise index of insulin sensitivity [30].

The control group consisted of 20 healthy women matched for glucose level (87.12 ± 3.29 mg/dL). They were 18–28 years old (mean ± 23), with regular menstrual cycles, no clinical or biochemical evidence of PCOS, and not treated with any drugs during the study and in the previous 3 months. In this group, normoglycemia was associated with normal weight (BMI 20–25, mean ± 23). Parameters chosen for control group rule out metabolic syndrome that negatively influences glucose metabolism.

Women who participated in the study were free of any systemic diseases, with no history of diabetes mellitus in the family. The blood for experiments was collected from women in the follicular phase of menstrual cycle.

### Laboratory analyses

Blood samples were collected in the morning from fasting patients. Each patient’s fasting insulin and blood glucose were measured, and intracellular deoxy-d-glucose transport into lymphocytes was assessed. Presence of glucose transporter GLUT4 in lymphocytes was also tested.

The blood tests were performed by using radioimmunoassay (RIA) and IRMA methods and were conducted in Endocrine Clinic of Medical Centre of Postgraduate Education. Measurements of intracellular deoxy-d-glucose transport and verification of GLUT4 presence in lymphocytes were performed in Chair of General Biology and Parasitology, Center for Biostructure Research, Medical University of Warsaw.

### Isolation of lymphocytes

Blood samples (8 mL) were collected into tubes with heparine. Lymphocytes were then isolated within next 2 h by means of centrifugation in Histopaque 1077 (Sigma–Aldrich) gradient, according to the manufacturers’ instructions. Isolated blood cells were washed twice in 0.9 % NaCl solution and then in transport solution (20 mM Hepes, 150 mM NaCl, 5 mM KCl, 5 mM MgSO4, 1.2 mM KH2PO4, 25 mM CaCl2, 2 mM pyruvic acid, pH 7.4) [18]. Cell density in medium was counted using the Bürker chamber. To investigate the uptake of deoxy-d-glucose, about 1 × 10^6^ cells/mL were suspended in transport solution.

### Lymphocytes viability control test

The test was conducted in order to verify the survival of the cells during the experiment. To 290 μL of cellular suspension (about 3 × 10^5^ lymphocytes), 1.5 μL of deoxy-d-glucose 2-[3H(G)] (8.0 Ci/mmol; Perkin Elmer) was added. After 60 min of incubation, 1 % solution of trypan blue was added at 1:1 volume ratio. The number of dead lymphocytes in a sample of 500 cells was counted using a light microscope.

### Intracellular transport of deoxy-d-glucose into lymphocytes

The study was carried out according to the method described by Kaliman et al. [18] with a partial modification [32]. To 290 μL of cellular suspension (about 3 × 10^5^ lymphocytes), 1.5 μL of deoxy-d-glucose 2-[3H(G)] (8.0 Ci/mmol; Perkin Elmer) and 7.5 μL of PBS were added. In order to evaluate the non-specific uptake of tritium-labeled deoxy-d-glucose (time t0), 7.5 μL of the “stop solution” (50 mmol/L of d-glucose in PBS) was added to the cellular suspension instead of the PBS solution. In order to investigate the dynamics of deoxy-d-glucose uptake by lymphocytes, the incubation time with isotope equaled 15, 30, and 60 min. After this time, (299 μL) 200 μL of cold “stop solution” (4 °C) was added to the sample. The cells were then rinsed three times by centrifugation in the “stop solution” (1,800 rpm, 10 min, 4 °C). After the centrifugation, the cells (about 3 × 10^5^ lymphocytes) were lyzed by adding 50 μL of lyzing solution (1.1 mmol/L NaOH; 0.1 % SDS). The cells have been lysed for 24 h. Afterward, 25 μL of lysed cells (about 1.5 × 10^5^ lymphocytes) were evaluated for the amount of tritium-labeled deoxy-d-glucose uptake. This procedure was repeated for each time point (15, 30, and 60 min) for each woman (*n* = 40).

The amount of deoxy-d-glucose uptake was measured with a liquid scintillation counter (Microbeta Trilux, Wallac, Finland). Radioactivity was evaluated in cpm. The label uptake was assessed basing on results of total label accumulated at the given time minus the non-specific uptake of deoxy-d-glucose. The non-specific uptake of deoxy-d-glucose was evaluated after incubating cells in “stop solution” instead of PBS. Many laboratories employ this method as a model system for investigating glucose transport [15, 19].

### Immunocytochemistry

The presence of GLUT4 (in intracellular compartment and on plasma membrane) in lymphocytes was investigated. The isolated lymphocytes (about 5 × 10^4^ from patients and control group) were set up in different slides and dried for 2 h at room temperature. Endogenic peroxidase has been blocked by adding 200 μL of 3 % H_2_O_2_ solution to the isolated cells for 10 min. The cells were then rinsed 3 times in PBS. Afterward, the lymphocytes were placed for 30 min in a blocking buffer [1 % bovine serum albumin (BSA) in PBS] with 2 % goat serum (Sigma). For the investigation of GLUT4 proteins, lymphocytes were incubated with rabbit polyclonal antibody against GLUT4 (Chemicon International Inc. Ca) diluted 1:100 in the blocking buffer. The used antibody is aimed against intracellular C-terminus of human’s proteins. Using this antibody allows visualization of GLUT4 in whole cells. Horseradish peroxidase-conjugated goat anti-rabbit IgG (Chemicon International Inc. Ca) at the concentration of 1:2,000 (diluted in the blocking buffer) was used as a secondary antibody. After each incubation period, the cells were rinsed. The antigen–antibody complex was visualized using DAB according to the manufacturer’s instructions (Sigma). The negative control (for exclusion of non-specific binding of antibodies) consisted of the lymphocytes incubated without the first antibody. The presence of GLUT4 was assessed in 100 cells for 3 times (total of 300 cells) using a light microscope (1,000 ×). The microscopic images were saved to a computer.

### Presentation of results

The uptake of deoxy-d-glucose by lymphocytes in 3 studied time points (15, 30, and 60 min) was presented as cpm average values ± SD. We also determined the changes in growth rate of uptake of deoxy-d-glucose in following time points. These results are presented as a profile of deoxy-d-glucose uptake (d cpm). To this end, the cpm value obtained for 15 min of the experiment was described as “1”. Next, cpm value for 30 and 60 min was divided by cpm value obtained in 15 min. Obtained d cpm values showed changes in growth rate in the following time points. Analogically to the uptake of deoxy-d-glucose, the result of the profile is presented as d cpm ± SD values.

Cpm and d cpm values were calculated for each of the patients and of the control women.

### Statistical analysis

Obtained results concerning the intensity of intracellular transport of deoxy-d-glucose into lymphocytes were compared using nonparametric Wilcoxon’s test with 95 % confidence interval (*p* = 0.05).

## Results

### Lymphocytes viability control test

Lymphocytes viability control test demonstrated that the applied research method does not have a significant impact on cell survival. A similar percentage of dead lymphocytes (about 5 %) were observed in individual samples. No differences in surveillance, comparing lymphocytes from healthy and PCOS women, were found.

### Intracellular transport of deoxy-d-glucose into lymphocytes

**Fig. 1:**
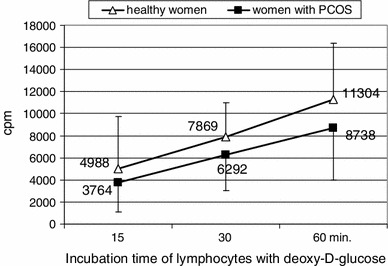
The intensity of deoxy-d-glucose uptake by lymphocytes of healthy women (average of 20 healthy women’s results) and PCOS patients (average of 20 PCOS patients’ results)

Figure 1 demonstrates intensity of deoxy-d-glucose uptake by lymphocytes (cpm). In case of lymphocytes from PCOS women, the cpm value in all studied time points was lower as compared to the cpm value for lymphocytes of healthy women. The observed differences in the cpm values between particular samples were statistically significant (*p* < 0.05).

**Fig. 2:**
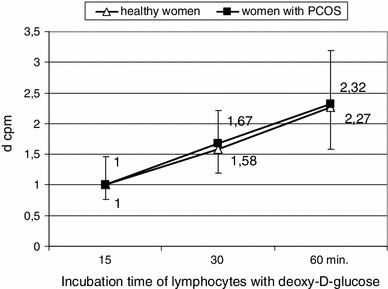
Profile of deoxy-d-glucose uptake by lymphocytes of healthy women and PCOS patients

Figure 2 demonstrates profile of deoxy-d-glucose uptake (d cpm). It shows that in 30 min of experiment glucose uptake increased about 1.5 times and in 60 min over 2 times in comparison with 15 min. These results are similar in healthy as well as in PCOS patients. Comparing value of d cpm observed in particular time points for lymphocytes from patients with PCOS and healthy women, no significant differences were found; thus, the pattern of deoxy-d-glucose uptake is similar in both investigated groups of women.

### Immunocytochemistry

**Fig. 3:**
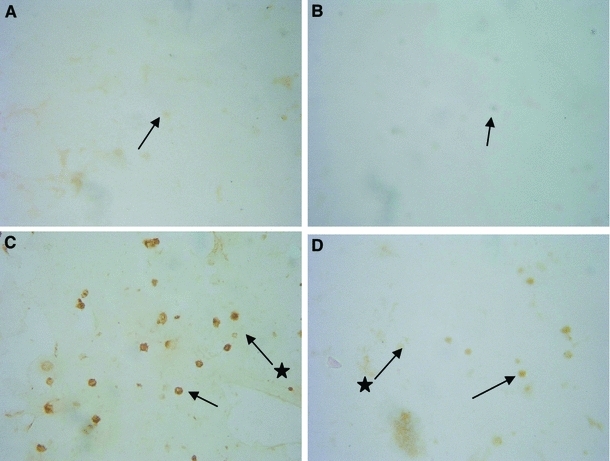
Immunocytochemical detection of GLUT4 in lymphocytes. **a** negative control for one of the women with PCOS (lymphocytes incubated without the first antibody). **b** negative control for one of the healthy women (lymphocytes incubated without the first antibody) The *arrow* shows an exemplary negative cell. **c** lymphocytes with positive reaction against GLUT4 from one of the PCOS patient. **d** lymphocytes with weak positive reaction against GLUT4 from one of the healthy women (in lymphocytes of other investigated healthy women, immunocytochemical reaction against GLUT4 was negative). The *arrow* shows an exemplary lymphocyte with positive reaction against GLUT4. The *arrow* with a *star* shows an exemplary lymphocyte without detected positive reaction against GLUT4

Negative control (the cells to which the first antibody was not added), performed in order to determine the presence of GLUT4 proteins, did not return color reaction in any of the samples (Fig. 3a, b). In the case of lymphocytes of PCOS women, in samples of 18 patients, most cells were stained brown, denoting the presence of GLUT4 (Fig. 3c). When lymphocytes of healthy women were investigated, a small number of light-colored cells were present only in 2 samples (Fig. 3d).

## Discussion

The obtained results indicate that in lymphocytes of PCOS women with normal plasma glucose, the intensity of deoxy-d-glucose uptake is decreased in comparison with healthy subjects. Presumably, this fact could be caused by impaired glucose transporters typical for lymphocytes.

In lymphocytes of patients with diabetes and in lymphocytes incubated in high concentration of glucose in medium, GLUT4 is also expressed [31, 33]. In our study, we investigated the presence of GLUT4 and we showed that GLUT4 is also present in lymphocytes of PCOS women with normoglycemia.

Decreased deoxy-d-glucose uptake in PCOS patients may indicate that the amount of functioning transporters on plasma membrane was changed compared to healthy subjects. One of the possibilities is that GLUT4 in lymphocytes of investigated PCOS patients was synthesized in order to try to compensate glucose uptake that was decreased. The attempt to compensate glucose level may succeed if GLUT4 would translocate from intracellular compartment to plasma membrane. As deoxy-d-glucose uptake was decreased, GLUT4 was probably not translocated to plasma membrane.

There were no significant differences in the pattern of deoxy-d-glucose uptake in PCOS patients compared with healthy women. This suggests that the capacity to deoxy-d-glucose uptake was maintained in investigated time points.

There is a possibility that insulin resistance is another cause of decreased uptake of glucose. Insulin resistance is present in 50–90 % of patients with PCOS [24, 25]. This condition is assessed using HOMA-IR and QUICK indices. The level of HOMA-IR was higher in obese PCOS group than in the control group consisting of obese patients without PCOS [39]. Although the HOMA-IR has been widely used, its cutoff for insulin resistance has not been conclusive [12]. In the study on non-diabetic, normotensive individuals, the cutoff value of HOMA-IR was 1.8 [12]. In the other study, HOMA-IR > 2.0 was used for screening test for glucose intolerance in PCOS women [37]. In conditions such as diabetes, glucose intolerance and others associated with insulin resistance, QUICK index values have been observed to be lower when compared to those of healthy volunteers [30]. Adult patients with a QUICK index below 0.375 tend to have higher risk of metabolic syndrome [16]. HOMA-IR and QUICK indices of PCOS patients in our study were upper limit of normal (HOMA–IR = 1.99 ± 1.17; QUICK = 0.350 ± 0.03) which in future may lead to insulin resistance that influences negatively uptake of glucose.

Studies show that even lean women with PCOS have elevated insulin resistance when compared with findings in body mass index-matched controls [25]. It was revealed also that insulin resistance in PCOS is present regardless of body mass index, but potentialized by obesity [9]. BMI of PCOS women participated in our study differs significantly (*p* < 0.05) from control group. Owing to papers of Messer et al. and Dantas et al., it seems that overweight in PCOS is not a key factor that influences insulin resistance.

There is no data considering glucose metabolism in lymphocytes of women with PCOS. It is also unclear whether PCOS is associated with changes in quality and quantity of GLUT proteins in lymphocytes. The obtained results do not provide clear answer why decreased intensity of intracellular deoxy-d-glucose transport into peripheral blood lymphocytes is present among PCOS patients with normoglycemia. In this case, further studies are necessary to evaluate insulin resistance as well as glucose transporter content in the studied blood cells.

The obtained results indicate that PCOS significantly decreased intensity of deoxy-d-glucose uptake into lymphocytes but did not change the pattern of deoxy-d-glucose uptake in cells of PCOS women with normal plasma glucose. The difference between amount of GLUT4 in lymphocytes of PCOS women and healthy subjects represents an increased expression of protein due to increased synthesis or decreased catabolism.
